# Cardiometabolic Changes in Different Gonadal Female States Caused by Mild Hyperuricemia and Exposure to a High-Fructose Diet

**DOI:** 10.1155/2018/6021259

**Published:** 2018-08-28

**Authors:** J. Soutelo, Y. A. Samaniego, M. C. Fornari, C. Reyes Toso, O. J. Ponzo

**Affiliations:** ^1^Department of Physiology, Medicine School, University of Buenos Aires (UBA), Buenos Aires, Argentina; ^2^Endocrinology Service Medical Complex, Argentine Federal Police (PFA), Churruca-Visca Hospital, Buenos Aires, Argentina; ^3^Laboratory Fornari-Bioalpha, Buenos Aires, Argentina

## Abstract

**Background:**

The objective of this study is to observe if mild hyperuricemia and a high-fructose diet influence the cardiovascular and metabolic systems in hypogonadic female Wistar rats compared to normogonadic female rats.

**Methods:**

Fifty-six (56) adult female Wistar rats were used in the present work. Animals were divided into two groups: normogonadic (NGN) and hypogonadic (HGN). These groups were also divided into four subgroups in accordance with the treatment: control with only water (C), fructose (F), oxonic acid (OA), and fructose + oxonic acid (FOA). Lipid profile, glycemia, uric acid, and creatinine determinations were assessed. Cardiovascular changes were evaluated by measuring blood pressure, myocyte volume, fibrosis, and intima-media aortic thickness.

**Results:**

HGN rats had higher levels of total cholesterol (TC) (*p* < 0.01) and noHDLc (*p* < 0.01), in addition to higher levels of uric acid (*p* < 0.05). The OA group significantly increased myocyte volume (*p* < 0.0001) and the percentage of fibrosis as well as the group receiving FOA (*p* < 0.001) in both gonadal conditions, being greater in the HGN group. Hypogonadic animals presented a worse lipid profile.

**Conclusion:**

Mild hyperuricemia produces hypertension together with changes in the cardiac hypertrophy, fibrosis, and increased thickness of the intima media in hypogonadic rats fed high-fructose diet.

## 1. Introduction

Cardiovascular disease (CVD) remains the leading cause of death in women. Apart from age, menopause status increases the prevalence of cardiometabolic risk factors as obesity, metabolic syndrome, type 2 diabetes, and hypertension [1, 2], while the exact mechanisms remain unclear. On the one hand, estrogen decreases and relative hyperandrogenism could bring on changes in the body composition, with an increment in the overall fat mass, with predominance of visceral fat and ectopic fat storage (liver and skeletal muscle), as well as a peripheral fat (gluteofemoral) decrease. These conditions would lead to an increment of proinflamatory adipocytokines and a state of insulin resistance [3]. Likewise, the estrogen decrease and the insulin resistant condition generate an atherogenic lipid profile with modified LDLs, increment of VLDL and triglycerides, and HDLc decrease [4].

Hypertension is a significant risk factor of CVD in women [5]. The involved mechanisms include some of the factors previously mentioned. Otherwise, an increase of uric acid levels in menopausal women has been observed. Usually, estrogens increase the uric acid excretion due to its uricosuric action; the estrogen decrease and the insulin resistance reduce the uric acid clearance, favoring hyperuricemia [6]. In a prospective study [7] over 50 years of follow-up, higher levels of serum uric acid increased the risk of gout in a graded manner among women, but the rate of increase was lower than that among men. Increasing age, obesity, hypertension, alcohol consumption, and diuretic use are also associated with the risk of incidence of gout among women. Therefore, the uric acid and gout have proved to be relevant in cardiovascular diseases [8]. Over the last decade, there were many significant advances in cardiovascular disease for women, including sex-specific research, but there is still more to be clarified.

The objective of this study was to observe if mild hyperuricemia and a high-fructose diet influence over the cardiovascular and metabolic systems in hypogonadic female Wistar rats compared to normogonadic female rats.

## 2. Materials and Methods

### 2.1. Animals

Fifty-six female adult Wistar rats from the Department of Physiology, School of Medicine, University of Buenos Aires, were used in this experiment. Animals were housed in a light-, temperature-, and humidity-controlled environment (lights on from 07:00 am to 07:00 pm, T: 22–24°C) and were fed *ad libitum*, having access to water during the entire experiment. When animals began the experiment, they were 70 days old. Animal handling and experiments were performed according to the “Ethical Principles and Guidelines for Experimental Animals” of the Swiss Academy of Medical Sciences (3rd Edition 2005).

#### 2.1.1. Experimental Design

Eight groups of adult female Wistar rats (*n* = 7/group) were studied over a period of 5-week treatment: four normogonadics (NGN) and four hypogonadics (HGN). The NGN group (70 days old) was divided into four subgroups: (a) control group (C): fed standard commercial diet and water; (b) fructose group (F): fed the same diet plus 10% (*w*/*v*) fructose (Tate & Lyle, USA) in drinking water, according to previous studies [9]; (c) oxonic acid group (OA) (Sigma Aldrich 156124, St. Louis, MO, USA): fed standard commercial diet and water and receiving the uricase inhibitor OA by intragastric gavage (750 mg/kg BW, daily) [9, 10]; and (d) fructose and oxonic acid group (FOA): fed control diet plus 10% (*w*/*v*) fructose in drinking water and receiving also the oxonic acid by intragastric gavage (750 mg/kg BW, daily), during the entire period.

In the second group (HNG), adult female rats underwent surgical procedures with an aseptic technique. The ovariectomized rats were anesthetized with an injection of sodium pentobarbital (40 mg/kg; intraperitoneal), and the bilateral ovaries were removed. Three weeks after ovariectomy (90 days old), the HNG animals were conducted in the experimental period and were divided into the same four subgroups receiving the same treatment as the four NGN groups: (a) control group (C); (b) fructose group (F); (c) oxonic acid group (OA); and (d) fructose and oxonic acid group (FOA) (Table 1).

All the animals that received fructose were individually housed in cages to calculate the water consumption per rat. In order to have the same level of stress caused by the gavage, animals in all control and fructose groups without OA received water administered by intragastric tube as a vehicle.

### 2.2. Body Weight and Systolic Blood Pressure Measurements

Body weight was measured daily. Amount of beverage consumed in each group of rats was calculated and adjusted daily according to the volume of liquid consumed. Systolic blood pressure (SBP) was measured in conscious rats by a validated volume-based tail-cuff method connected to an amplifier and a data acquisition system (Rat Tail System; Innovators in Instrumentation, Landing, NJ, USA). All animals were preconditioned for blood pressure measurements 1 week before each experiment. SBP was measured basal, 2nd week, and 4th week. Before the measurements, rats were placed in a holder preheated to 35°C. An average value from three SBP readings (that differed by no more than 2 mmHg) was determined for each animal after they had become acclimatized to the experimental environment.

### 2.3. Blood Measurements

At the end of the 5-week treatment period, all animals were sacrificed between 9:00 and 10:00 am by decapitation. NGN animals were sacrificed at diestrus phase. Trunk blood samples were collected to measure plasma glucose, creatinine, uric acid, and lipid profile total cholesterol (TC), triglycerides (TG), and HDL cholesterol (HDLc); TG/HDL cholesterol index was calculated as a surrogate marker of insulin resistance (IR) [11], and no-HDLc was calculated as TC minus HDLc. All these values were assayed by commercial kits implemented in an automated clinical analyzer. The estradiol assay was performed by a chemiluminescent microparticle immunoassay (CMIA) (Architect, Abbott Longford Co., Ireland).

### 2.4. Cardiovascular Outcomes

#### 2.4.1. Morphometric Determination of Myocyte Size

Cardiomyocyte sizes were measured on hematoxylin-eosin- and PAS-stained sections. In order to have a consistent result, myocytes positioned perpendicularly to the plane of the section with a visible nucleus and cell membrane clearly outlined and unbroken were selected for the cross-sectional area measurements. Myocyte volume (myocyte hypertrophy) was calculated from individual myocyte area (formula: length (*μ*m) × width (*μ*m) × 7.59 × 10^−3^) based on the previously demonstrated correlation between these parameters [12]. A total of 50 myocytes were selected per animal from the left ventricle of each heart and analyzed by a blinded observer to the experimental treatment.

#### 2.4.2. Fibrosis

Sections were stained with Masson's trichrome. Positive blue color was analyzed using ImagePro Plus (Media Cybernetics).

#### 2.4.3. Intima Media of the Aorta

At the end of the experiment, the thoracic aorta (from the arch to the diaphragm) was harvested, cut in half, and either fixed in buffered formalin or snap frozen. Aorta rings were embedded in paraffin, and 4 *μ*m sections were cut and stained with hematoxylin and eosin (HE). Quantification of injured area in HE-stained aorta sections was performed using ImagePro Plus software and analyzed by a blinded observer to the experimental treatment.

#### 2.4.4. Arteriole

For each arteriole, the outline of the vessel and its internal lumen (excluding the endothelium) were generated using computer analysis (ImagePro Plus 7.0; Media Cybernetics) to calculate the total arteriolar medial area (outline/inline) in 30 arterioles per group. The media/lumen (M/L) ratio was calculated by the outline/inline relationship.

### 2.5. Statistical Analysis

Values are expressed as means ± SEM. Significant differences between treatment groups were determined by two-way ANOVA. When *p* < 0.05, ANOVA posttest comparisons were made using a Bonferroni multiple comparison test. The relationship between variables was assessed by correlation analysis. Statistical analysis was performed using Prism version 5.04 (GraphPad Software, San Diego, CA). Also, data were analyzed by general linear model, which in addition introduces the interaction between the factors in the model and transforms the heterogeneity of variances when, even with transformations, normality and homogeneity of variances are achieved. When a variable was observed over time, such as SBP, a random factor was introduced (hence, the mixed denomination, the presence of fixed factors, treatments and the gonadal condition, and a random factor (time)). In order to analyze the differences of each random variable between the treatments and the gonadal state, the multiple comparisons method of Di Rienzo, Guzman, and Casanoves (DGC) was used, using the multivariate cluster analysis technique [13].

## 3. Results

### 3.1. Body Weight

All NGN animals began the experiment with similar weights (*p* = NS), and at the end of the experiment, a significant increase was observed between all groups (*p* < 0.0001). The C and F groups were heavier than the OA and FOA groups (*p* < 0.001) (Table 2).

All HGN animals began the experiment with similar weights (*p* = NS), and at the end of the experiment, a significant increase was observed in the F and FOA groups (*p* < 0.0001) when compared to the rest of the groups.

HGN animals began the experiment with a significant higher weight than the NGN animals (*p* < 0.0001). But at the end of the experiment, a significant difference was observed only between C groups (Table 2).

### 3.2. Water Intake

NGN animals that received F and FOA drunk more water than the rest of the groups (F: 66.70 ± 3.30 mL/day/animal and FOA: 47.57 ± 2.50 versus C: 31.80 ± 2.50; OA: 30.34 ± 1.00; *p* < 0.001). Similar results were observed in the HGN groups (F: 124 ± 4.50; FOA: 109 ± 3.50 versus C: 31 ± 1.00 and OA: 30 ± 1.00; *p* < 0.001). The HGN F and FOA groups drank more than the NGN F and FOA groups (*p* < 0.01).

### 3.3. Blood Pressure

#### 3.3.1. In Normogonadic Animals

**Figure 1:**
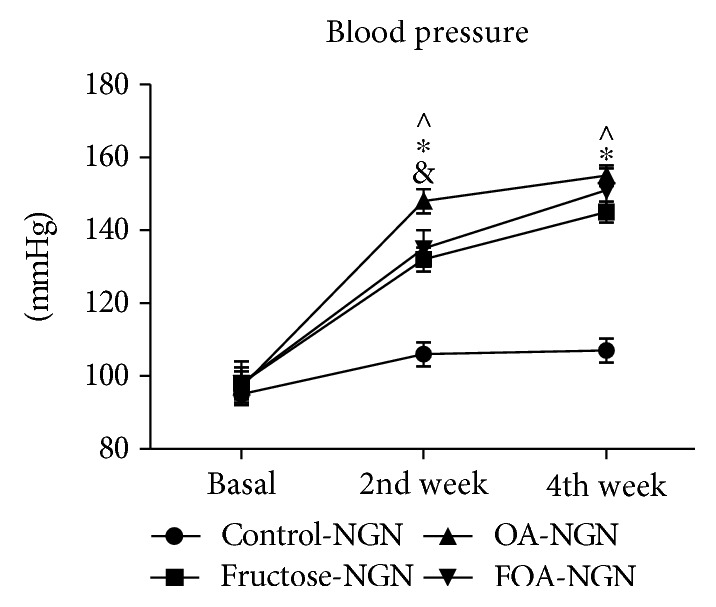
Systolic blood pressure in NGN groups at basal time, 2nd week, and 4th week after the beginning of treatment. NGN: normogonadic; OA: oxonic acid; FOA: fructose and oxonic acid. ^&^*p* < 0.05 control versus FOA group at 2nd week. ^∗^*p* < 0 001 control versus fructose group at 2nd week and 4th week and OA versus FOA group at 2nd week. ^∧^*p* < 0.0001 control versus OA group at 2nd week and 4th week and control versus FOA group at 4th week.

The C group did not show SBP changes during the experiment. At the end of the experiment, SBP was significantly higher in all treatments versus C group (*p* < 0.0001) (Figure 1).

#### 3.3.2. In Hypogonadic Animals

**Figure 2:**
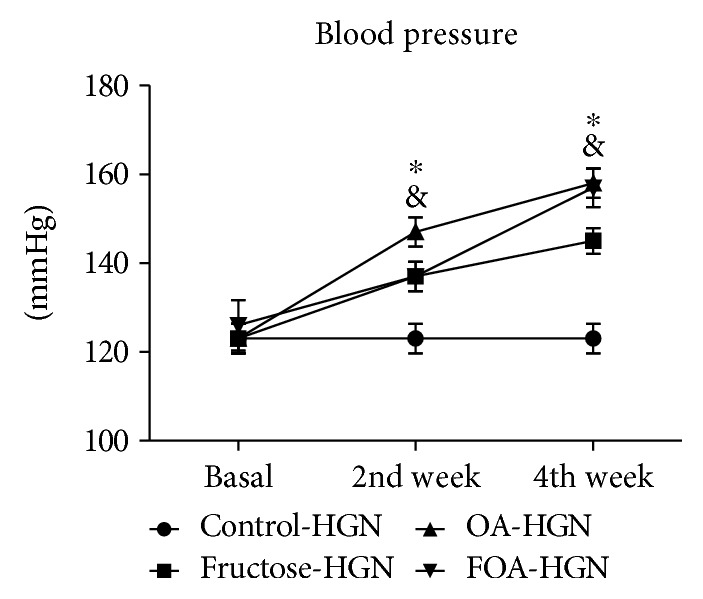
Systolic blood pressure in HGN groups at basal time, 2nd week, and 4th week after beginning of treatment. HGN: hypogonadic; OA: oxonic acid; FOA: fructose and oxonic acid. ^&^*p* < 0.05 control versus OA group at 2nd week and control versus fructose group at 4th week. ^∗^*p* < 0.001 control versus OA and FOA group at 4th week.

The C group showed a significant (*p* < 0.01) increment in SBP during the experiment. At the end of the experiment, SBP was significantly higher in all treatments versus C group (*p* < 0.001) (Figure 2).

When compared by gonadal condition, significant differences were found in the C group at basal time and during the entire experimental time (Table 3).

### 3.4. Biochemical Variables

As expected, plasmatic estradiol decreased at a very low level in all hypogonadic animals (20.70 ± 0.30 pg/mL) compared to all ovariectomized rats (38.81 ± 2.86 pg/mL) (*p* < 0.0001). In both NGN and HGN groups, there was no difference in plasmatic creatinine levels when comparing treatment groups. No significant differences were observed in fasting glucose levels in the NGN groups. But in the HGN group, animals treated with F and FOA had higher glucose levels than in the C group (C: 94 ± 3.25 mg/dL; F: 104 ± 3.5 mg/dL; FOA: 106 ± 1.4 mg/dL; *p* < 0.05) (Table 4).

Uric acid (UA) levels were similar in all NGN animals. However, UA levels were significantly higher in HGN animals treated with OA (1.64 ± 0.10 mg/dL) and FOA (1.63 ± 0.08 mg/dL), when comparing them to their respective C group (1.06 ± 0.06 mg/dL) (*p* < 0.001). Moreover, there were significant differences when comparing NGN and HGN animals for all treatments, being UA higher in HGN female rats (*p* < 0.01) (Table 4).

Regarding the lipidic profile, in NGN animals, no significant differences were found between the different experimental groups (Table 5). When comparing the effect of the gonadal condition, a significant increase in the plasmatic TC levels (*p* < 0.001) and no-HDLc (*p* < 0.001) was found in HGN female rats versus NGN all groups. In HGN animals, the F group showed a significant increase in the index TG/HDL (*p* < 0.05) when compared to the respective control. Likewise, in the same gonadal condition, the FOA group showed higher TC, triglycerides, no-HDLc levels, and TG/HDL index regarding the control group (*p* < 0.05). (Table 5). An inverse correlation was found between estradiol and TC levels (*r* = −0.50; *p* = 0.0006) and estradiol and no-HDLc (*r* = −0.32; *p* = 0.03).

### 3.5. Cardiovascular Histology

#### 3.5.1. Morphometric Determination of Myocyte Size

**Figure 3:**
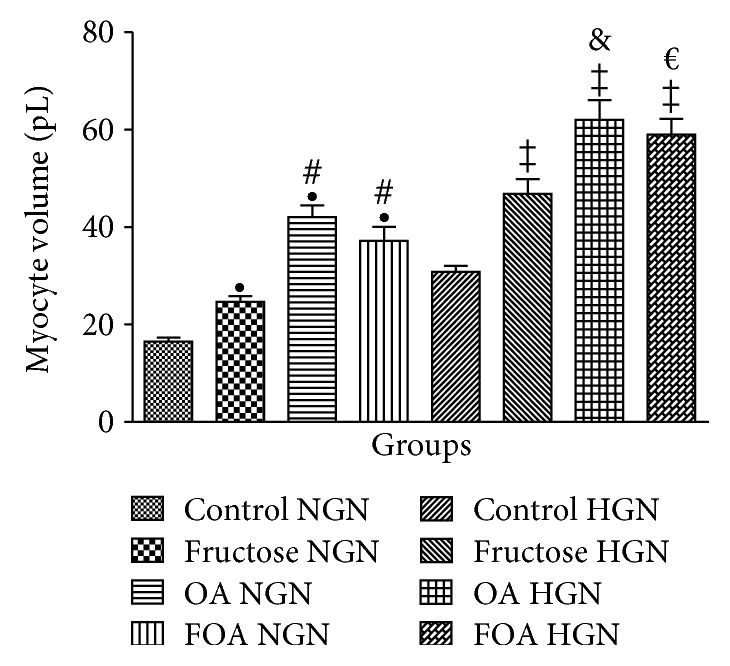
Myocyte volume (pL). NGN: normogonadic; HGN: hypogonadic; OA: oxonic acid; FOA: fructose and oxonic acid. ^•^*p* < 0.0001 fructose, OA, and FOA NGN versus control NGN group. ^#^*p* < 0.0001 OA and FOA NGN versus fructose NGN group. ^‡^*p* < 0.0001 fructose, OA, and FOA HGN versus control HGN group. ^&^*p* < 0.01 OA HGN versus fructose HGN group. ^€^*p* < 0.05 FOA HGN versus fructose HGN group.

In the NGN group, animals treated with OA showed greater volume, followed by the ones treated with FOA and F regarding the C group (*p* < 0.001). In the HGN group, the same pattern was observed as in NGN animals. We found significant differences when we analyzed the different NGN versus HGN groups receiving the same treatment (*p* < 0.0001) (Figure 3).

#### 3.5.2. Fibrosis

**Figure 4:**
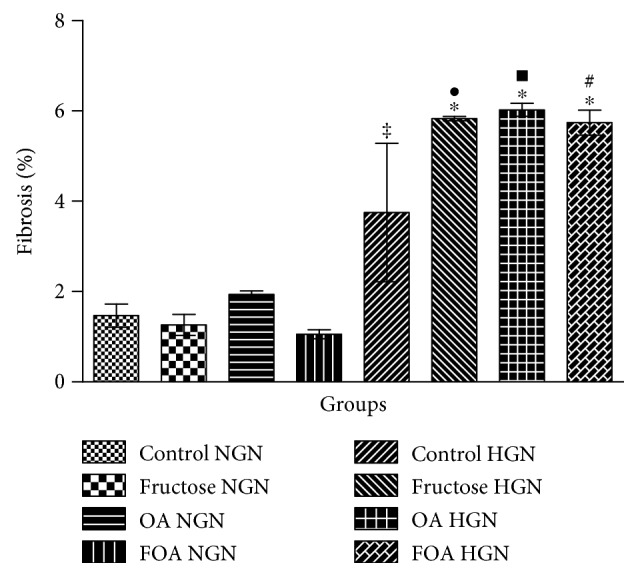
Percentage of cardiac fibrosis. NGN: normogonadic; HNG: hypogonadic; OA: oxonic acid; FOA: fructose and oxonic acid. ^∗^*p* < 0.001 F, OA, and FOA HGN versus control HGN group. ^‡^*p* < 0.0001 control HGN versus control NGN group. ^•^*p* < 0.0001 fructose HGN versus fructose NGN group. ^■^*p* < 0.0001 OA HGN versus OA NGN group. ^#^*p* < 0.0001 FOA HGN versus FOA NGN group.

Significant increment of fibrosis percentage was only found in HGN female rats in the F, OA, and FOA groups when compared to the C group (*p* < 0.001). Likewise, significant differences were found between NGN and HGN groups receiving similar treatments (*p* < 0.0001) (Figure 4).

#### 3.5.3. Intima Media of the Aorta

**Figure 5:**
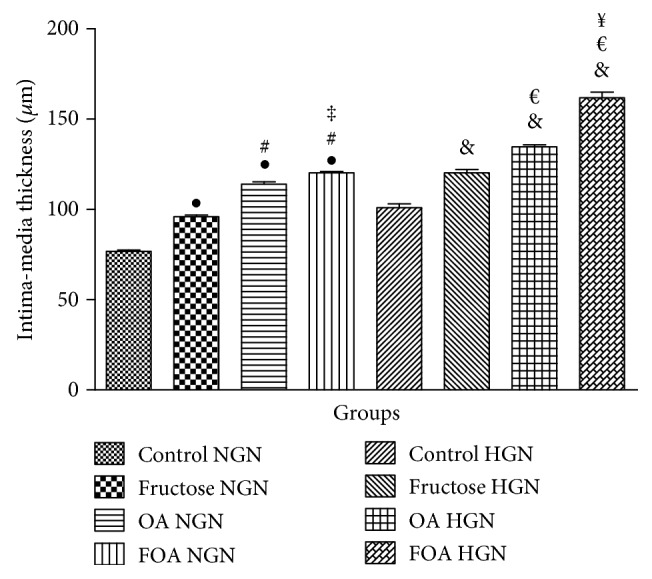
Intima media thickness. NGN: normogonadic; HNG: hypogonadic; OA: oxonic acid; FOA: fructose and oxonic acid. ^•^*p* < 0.0001 fructose, OA, and FOA NGN versus control NGN group. ^#^*p* < 0.0001 OA and FOA NGN versus fructose NGN group. ^‡^*p* < 0.0001 FOA NGN versus OA NGN group. ^&^*p* < 0.0001 fructose, OA, and FOA HGN versus control HGN group.^€^*p* < 0.0001 OA and FOA HGN versus fructose HGN group. ^¥^*p* < 0.0001 FOA HGN versus OA HGN group.

**Figure 6:**
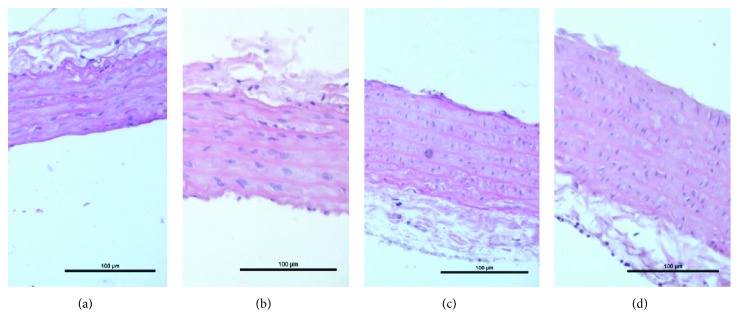
Intima media of the Aorta. HE (10x). (a) NGN control. (b): NGN FOA. (c) HGN control. (d) HGN FOA.

In both gonadal conditions, the intima media was significantly thicker in the FOA (*p* < 0.001), OA (*p* < 0.001), and F (*p* < 0.001) groups when compared to C animals. Moreover, HGN animals showed a thicker intima media when compared to NGN animals receiving the same treatment (*p* < 0.0001) (Figures 5 and 6).

#### 3.5.4. Arteriole Media/Lumen (M/L) Ratio

In NGN animals, we observed a greater arteriolar M/L ratio in the OA group (*p* < 0.01) and the FOA group (*p* < 0 001) when compared to the C group. In the F group, we found a strong trend to the increase of this ratio, but not reaching a statistically significant difference. In the HGN groups, no significant differences were found between the different treatments. However, significant differences were found between the NGN and HGN groups receiving similar treatment (Table 6).

## 4. Discussion

Our main findings can be summarized as follows: (1) more elevated blood pressure was observed in HGN rats when compared to NGN rats, being greater in the animals treated with OA and FOA of both gonadal conditions; (2) abnormal myocardial architecture: enlarged and cardiac fibrosis were significantly greater in the HGN animals of the OA and FOA groups; (3) HGN animals showed a thicker intima media of the aorta and a greater arteriolar media/lumen ratio, when compared to NGN animals receiving the same treatment, being greater in the animals treated with OA and FOA; (4) the HGN rats presented an atherogenic lipid profile; furthermore, the FOA group showed the highest TG/HDL ratio; (5) finally, uric acid was significantly higher in the OA and FOA groups and glycemia was higher in the FOA and F groups of HGN rats.

The estrogen deficiency per se causes hypertension [13]. In this setting, data demonstrate that about 50% of postmenopausal women experience moderate to severe hypertension or take antihypertensive drugs [14]. Estrogen inhibits the renin-angiotensin-aldosterone system (RAAS), through reduction of the expression of the angiotensin 1 receptors (AT1) and suppresses endothelin, a strong vasoconstrictor [14, 15]. Moreover, Huber et al. demonstrated in a menopausal rat model that long-term estrogen deficiency produces a dysfunction in the cardiovascular autonomic response, favoring hypertension development [15]. In addition, estrogen deficiency induces endothelium dysfunction [16, 17]. Likewise, uric acid levels also may be associated with hypertension, which in turn has been associated with endothelial dysfunction. Zoccali et al. demonstrated an association between uric acid and cardiovascular disease, particularly prominent in postmenopausal women [18]. Endogenous estradiol plays a role in preserving endothelial function and in lowering serum uric acid (SUA) level independent of cardiovascular risk factors. In menopause, decreased estrogen levels and increased SUA levels may promote endothelial dysfunction and development of hypertension [19].

Estrogen is known to have multiple protective effects in cardiovascular function [20]. Cardiomyocytes and cardiac fibroblasts contain estrogen receptor isoforms (ER*α* and ER*β*). Estrogen acts through binding ER*α* and ER*β* receptors, which are able to mediate the stimulation of the phosphatidylinositol 3-kinase (PI3K) and protein kinase B (Akt) pathway [21]. Moreover, the prosurvival protein Akt also activates the Bcl-2-related antiapoptotic pathway [21]. Several studies have shown that deficiency of estrogen promotes cardiac apoptosis-related death, which may worsen cardiac dysfunction and lead to heart failure [22–24].

Hypertension causes pathological cardiac hypertrophy and is recognized as the most important predictor of cardiovascular morbidity and mortality, as well as an important risk factor for heart failure [25].

Inflammatory cytokines could be a key in the pathogenesis of hypertension and cardiovascular disease. TNF-*α* is a proapoptotic molecule and proinflammatory factor [26]. Additionally, estrogen deficiency produces endothelial damage and stimulates cytokines [5, 27].

In the present study, we observed that hyperuricemia, exposure to fructose, and the combination of both produce hypertension, intima-media aortic thickening, and myocardiocyte hypertrophy in NGN female rats. These results are more relevant in the absence of estrogens.

Several studies have been observed that, in postmenopausal women, hyperuricemia induces the increase of inflammatory markers [28, 29], favoring endothelial damage [30, 31], hypertension, and cardiac damage [32–35].

Postmenopausal women tend to worsen lipid profile which may explain the increased cardiovascular risk [36]. After menopause, TC and LDLc usually increase and these changes are accompanied by a decrease in HDLc and an increase in TG. Lower HDLc/TC and apo-AI/apo-B ratios, as well as direct association of small LDLc particles with high TG levels and inverse associations of HDLc, were reported following menopause [36]. Increased TG rich lipoproteins are associated with higher proportions of small dense LDLc. Small dense LDLc is more susceptible to oxidation, transendothelial transport, and deposition in artery wall [37].

Uric acid is a heterocyclic compound that is created when the body breaks down purine nucleotides. About 70% of daily uric acid excretion occurs via the kidneys, and in 5–25% of humans with impaired renal excretion leads to hyperuricemia. Hyperuricemia has been associated with acute and chronic diseases, including gout, cardiovascular disease, and type 2 diabetes, though these associations are typically observed among postmenopausal women. Mumford et al. demonstrated that estrogen and progesterone were found to increase the renal clearance of uric acid, thereby decreasing serum uric acid levels in regularly menstruating women [38]. Recently, Jung et al. showed that the administration of estrogen plus progesterone therapy in menopause women was associated with reduced serum uric acid levels; however, estrogen therapy or tibolone use reduces serum uric acid levels, but not significantly [39]. While the exact mechanism is not yet elucidated, it is known that urate contained within glomerular filtrate flows into the tubular epithelium via URAT1, a urate-lactate exchanger which is expressed on the luminal side of the tubular epithelium. Intracellular urate flows out from the tubular epithelium into the interstice and blood via GLUT9, a facilitated urate transporter expressed on the antiluminal side of tubular epithelium, or flows out into the glomerular filtrate via ABCG2, a urate efflux transporter expressed on the luminal side of the tubular epithelium. Estrogen suppresses the levels of Urat1, Glut9, and Abcg2 proteins [40].

The strength of our study is based on the fact that we have demonstrated that a mild hyperuricemia alone and in association with a high-fructose diet produce a cardiovascular impact that is worsened in a hypogonadal condition.

Our limitation was not being able to study in more profoundness the molecular mechanisms that would produce the effects by us described.

We are convinced that the results obtained in the present study will encourage the development of additional research related to the effects of the menopause state and other conditions (hyperuricemia and high-fructose diet) on cardiovascular and metabolic systems, as well as the possible mechanisms involucrated in this process.

## 5. Conclusion

Summarizing hyperuricemia, a high-fructose diet and hypogonadic state are considered risk factors in the development of cardiovascular disease and heart failure. FOA and OA treatments produce hypertension together with cardiac hypertrophy, fibrosis, and increased thickness of the intima media, being more important these changes in a hypogonadal animal model. Of course, further studies are required to clarify the mechanisms by which these effects are produced.

## Figures and Tables

**Table 1:** Characteristics of the animals at the beginning of the study.

Groups	Age at the beginning of experiment	Estrogen (pg/mL)
NGN	70 days old	38.81 ± 2.86
HGN	90 days old	20.70 ± 0.3^∗^

Estrogen levels were expressed as mean ± SEM. NGN: normogonadic; HNG: hypogonadic. ^∗^NGN versus HGN *p* < 0.001.

**Table 2:** Body weight of animal at the start and at the end of experiment.

Weight (g)	Beginning of experiment	End of experiment	*p*
*NGN*			
Control	217 ± 12.00	383 ± 2.00^∗^	0.0001
Fructose	197 ± 8.00	391 ± 2.50^#^	0.0001
OA	207 ± 4.50	361 ± 1.00^∗^^#^	0.0001
FOA	205 ± 5.00	364 ± 2.00^∗^^#^	0.0001
*HGN*			
Control	342 ± 14.00	363 ± 15.00^∗∗^^##^	0.002
Fructose	348 ± 10.00	381 ± 10.00^∗∗^	NS
OA	344 ± 10.00	361 ± 15.00^∗∗^^##^	NS
FOA	347 ± 10.00	380 ± 10.00^##^	0.03

Data are expressed as mean ± SEM. NGN: normogonadic; HNG: hypogonadic; OA: oxonic acid; FOA: fructose and oxonic acid. ^∗^Control NGN versus OA and FOA NGN *p* < 0.001. ^#^Fructose NGN versus OA and FOA NGN *p* < 0.001. ^∗∗^Fructose HGN versus OA and control *p* < 0.001. ^##^FOA HGN versus OA and control HGN *p* < 0.001.

**Table 3:** Systolic blood pressure (SBP). Comparative effect of the gonadal state at different stages of treatments.

SBP (mmHg)	NGN	HGN	*p*
*Basal*			
Control	95 ± 2.88	122 ± 1.48	<0.0001
Fructose	98 ± 6.00	124 ± 2.29	0.001
OA	97 ± 4.35	123 ± 2.85	0.0004
FOA	98 ± 4.40	126 ± 2.85	<0.0001
*2nd week*			
Control	106 ± 3.33	123 ± 3.33	0.0003
Fructose	131 ± 3.33	136 ± 2.36	NS
OA	148 ± 1.01	146 ± 3.16	NS
FOA	147 ± 1.01	146 ± 1.54	NS
*4th week*			
Control	107 ± 3.33	126 ± 2.02	0.0004
Fructose	145 ± 2.88	145 ± 1.01	NS
OA	155 ± 3.00	158 ± 2.40	NS
FOA	151 ± 1.01	156 ± 2.02	NS

Data are expressed as mean ± SEM. NGN: normogonadic; HNG: hypogonadic; OA: oxonic acid; FOA: fructose and oxonic acid.

**Table 4:** Biochemical variables. Comparative effect of the gonadal state at different stages of treatments.

	NGN	HGN	*p*
*Creatinine (mg/dl)*			
Control	0.44 ± 0.01	0.44 ± 0.02	NS
Fructose	0.39 ± 0.01	0.42 ± 0.01	NS
OA	0.37 ± 0.02	0.41 ± 0.01	NS
FOA	0.43 ± 0.02	0.41 ± 0.01	NS
*UA (mg/dl)*			
Control	1.02 ± 0.03	1.06 ± 0.06	NS
Fructose	1.03 ± 0.05	1.21 ± 0.05	0.049
OA	1.16 ± 0.11	1.64 ± 0.10^∗∧^	0.009
FOA	1.15 ± 0.12	1.63 ± 0.08^∗∧^	0.008
*Glycemia (mg/dl)*			
Control	91.17 ± 3.84	94 ± 3.25	NS
Fructose	89.83 ± 3	103.8 ± 3.53^“^	0.01
OA	89 ± 7.23	99.67 ± 2.45	NS
FOA	103.8 ± 4.61	106.2 ± 1.4^“^	NS

Data are expressed as mean ± SEM. NGN: normogonadic; HNG: hypogonadic; OA: oxonic acid; FOA: fructose and oxonic acid; UA: uric acid; NS: nonsignificant. ^∗^*p* < 0.001 HGN control versus HNG OA and FOA groups. ^∧^*p* < 0.01 HGN frutose versus HNG OA and FOA groups. ^“^*p* < 0.05 HGN control versus HGN fructose and FOA groups.

**Table 5:** Lipid profile. Comparative effect of the gonadal state at different stages of treatments.

	NGN	HGN	*p*
*Total cholesterol (mg/dl)*			
Control	53.15 ± 2.13	76.84 ± 2.40	<0.0001
Fructose	56.68 ± 2.13	72.16 ± 4.66	0.01
OA	58.77 ± 1.66	73.61 ± 2.25	0.0003
FOA	54.93 ± 2.22	91.96 ± 4.38^∗^^#^	<0.0001
*Triglyceride (mg/dl)*			
Control	50.13 ± 4.77	61.48 ± 10.28	NS
Fructose	73.27 ± 12.72	83.98 ± 6.06	NS
OA	57.50 ± 9.8	57.61 ± 7.53	NS
FOA	83.58 ± 17.36	101.20 ± 11.68^∗∗^^&^	NS
*HDL (mg/dl)*			
Control	36.49 ± 4.43	36 ± 1.03	NS
Fructose	33.77 ± 1.76	34.39 ± 1.08	NS
OA	31.69 ± 1.39	33.14 ± 0.70	NS
FOA	32.72 ± 1.77	33.67 ± 1.11	NS
*No HDL (mg/dl)*			
Control	20.33 ± 1.65	32.60 ± 1.66	0.0006
Fructose	22.93 ± 2.58	34.77 ± 1.88	0.003
OA	27.01 ± 1.61	39.14 ± 3.78	0.01
FOA	23.61 ± 1.84	46.67 ± 2.18^†^^∧^	<0.0001
*TG/HDL*			
Control	1.56 ± 0.21	1.41 ± 0.21	NS
Fructose	2.18 ± 0.34	2.04 ± 0.25	NS
OA	1.84 ± 0.35	1.37 ± 0.16	NS
FOA	2.45 ± 0.42	2.33 ± 0.19^“^	NS

Data are expressed as mean ± SEM. NGN: normogonadic; HNG: hypogonadic; OA: oxonic acid; FOA: fructose and oxonic acid; NS: nonsignificant. ^∗^*p* < 0.05 HGN fructose versus HGN FOA group. ^#^*p* < 0.01 HGN OA versus HGN FOA group. ^∗∗^*p* < 0.05 HGN control versus HGN FOA group. ^&^*p* < 0.05 HNG OA versus HGN FOA group. ^†^*p* < 0.05 HNG control versus HGN FOA group. ^∧^*p* < 0.05 HGN fructose versus HGN FOA group. ^“^*p* < 0.05 HGN control versus HGN FOA group.

**Table 6:** Arteriole media/lumen (M/L) ratio.

Groups	NGN	HGN	*p*
Control	0.53 ± 0.03	0.80 ± 0.02	0.0002
Fructose	0.54 ± 0.02	0.96 ± 0.09	0.05
OA	0.67 ± 0.03	1.07 ± 0.08	0.004
FOA	0.84 ± 0.07^∗∧^	1.13 ± 0.09	0.05

Data are expressed as mean ± SEM. NGN: normogonadic; HNG: hypogonadic; OA: oxonic acid; FOA: fructose and oxonic acid. ^∗^*p* < 0.01 control versus FOA group. ^∧^*p* < 0.01 fructose versus FOA group.

## Data Availability

The data used to support the findings of this study are available from the corresponding author upon request.
